# The First 110,593 COVID-19 Patients Hospitalised in Lombardy: A Regionwide Analysis of Case Characteristics, Risk Factors and Clinical Outcomes

**DOI:** 10.3389/ijph.2022.1604427

**Published:** 2022-05-11

**Authors:** Nicole Mauer, Greta Chiecca, Greta Carioli, Vincenza Gianfredi, Licia Iacoviello, Silvia Bertagnolio, Ranieri Guerra, Anna Odone, Carlo Signorelli

**Affiliations:** ^1^ Vita-Salute San Raffaele University, Milan, Italy; ^2^ European Observatory on Health Systems and Policies, Brussels, Belgium; ^3^ Department of Biomedical Sciences for Health, University of Milan, Milan, Italy; ^4^ Department of Epidemiology and Prevention, University of Insubria, Varese, Italy; ^5^ IRCCS Neuromed, Pozzilli, Italy; ^6^ World Health Organization, Geneva, Switzerland; ^7^ Department of Public Health, Experimental and Forensic Medicine, University of Pavia, Pavia, Italy

**Keywords:** COVID-19, SARS-CoV-2, epidemiology, Italy, Coronavirus disease, Lombardy, hospital records, hospital admission statistics

## Abstract

**Objectives:** To describe the monthly distribution of COVID-19 hospitalisations, deaths and case-fatality rates (CFR) in Lombardy (Italy) throughout 2020.

**Methods:** We analysed de-identified hospitalisation data comprising all COVID-19-related admissions from 1 February 2020 to 31 December 2020. The overall survival (OS) from time of first hospitalisation was estimated using the Kaplan-Meier method. We estimated monthly CFRs and performed Cox regression models to measure the effects of potential predictors on OS.

**Results:** Hospitalisation and death peaks occurred in March and November 2020. Patients aged ≥70 years had an up to 180 times higher risk of dying compared to younger patients [70–80: HR 58.10 (39.14–86.22); 80–90: 106.68 (71.01–160.27); ≥90: 180.96 (118.80–275.64)]. Risk of death was higher in patients with one or more comorbidities [1: HR 1.27 (95% CI 1.20–1.35); 2: 1.44 (1.33–1.55); ≥3: 1.73 (1.58–1.90)] and in those with specific conditions (hypertension, diabetes).

**Conclusion:** Our data sheds light on the Italian pandemic scenario, uncovering mechanisms and gaps at regional health system level and, on a larger scale, adding to the body of knowledge needed to inform effective health service planning, delivery, and preparedness in times of crisis.

## Introduction

The ongoing COVID-19 pandemic has affected over 331 million people worldwide as of 18 January 2022 [1]. First cases were reported in the province of Hubei, China, 2 years ago in early December 2019 [2]. Its subsequent rapid international spread culminated in the World Health Organization (WHO) declaring it a global pandemic on 11 March 2020 [3]. The region of Lombardy in Northern Italy was the earliest European area to become affected, with the first autochthonous COVID-19 case identified in Codogno, Lombardy on 21 February 2020 [4]. Since the beginning of the pandemic, Italy has registered over 8.7 million cases of COVID-19 and over 141,000 deaths [1]. The rapid spread of infection has had a significant impact on the Italian national health system, particularly due to the sudden increase in hospitalisations and the overwhelming demand for intensive care unit (ICU) beds throughout, to date, three epidemic waves [5, 6]. Inexperience in handling epidemic situations, particularly of a previously unknown pathogen, and the initial absence of diagnostics, personal protective equipment and poorly updated pandemic preparedness and response structures, substantially aggravated the epidemiological scenario, in particular during the first epidemic wave [7]. The Italian National Health System has been undergoing important changes over the years, including the increasing decentralisation of health care planning and decision making towards regional administrations since the 1990s and the progressive integration of publicly delivered health services with those offered by private health care providers since the global financial crisis [8]. These trends have contributed towards exacerbating regional differences and inequalities in access to health services, while also shaping the landscape of service provision in Italy. The public system has been underfunded, while the private health sector has primarily invested in expanding profitable health services such as diagnostic, outpatient and non-essential care, with limited capacity building for essential hospital, emergency, and intensive care services [9]. It is possible that organisational determinants in the management of the pandemic, as well as the unequal distribution of resources and the poor coordination of response mechanisms between regions may have worsened the saturation of the health system at national level.

To date, Lombardy has suffered one third of all COVID-related deaths in Italy, experiencing up to 13,500 hospitalisations and 550 deaths per day at the peak of the first epidemic wave in mid-April 2020 [10–12] and up to 9,000 hospitalisations and 210 deaths per day at the peak of the second epidemic wave in autumn 2020 [10, 11]. This trend appears to be related to multiple factors [13, 14]. Firstly, Lombardy is the region with the highest population density (420 inhabitants per km^2^) [15] in Italy and, being home to the large urban centre of Milan, predominantly hosts a population of commuters: over 50% of inhabitants move outside their municipality on a daily basis for work, thereby facilitating spread of infection [16]. The occurrence of the first European COVID-19 cases in this uniquely interconnected region at a time when the ability to recognise, diagnose and treat the condition was still limited, substantially hindered initial containment responses. Moreover, collective evidence has shown that older population groups are particularly vulnerable to developing a severe form of COVID-19, requiring both hospitalisation and supportive treatments [17, 18]. At 23.2%, Italy displayed the highest proportion of people aged 65 years or above in the European Union in 2020 (EU average 20.6%). While in the context of Lombardy, the median age of the population in 2020 was well above the EU average at 47 years (EU average 43.9 years) [19, 20]. Lastly, Italy’s and Lombardy’s hospitals, were not fully prepared to handle shocks prior to the pandemic, with only 9 ICU beds per 100,000 inhabitants compared to a European average of 13 ICU beds [21].

In this context, gaining a clearer picture of the local dynamics of the epidemic, including identifying and stratifying risk factors, is crucial for policy makers to improve management in real-time and to inform the national and international Public Health community on how to better prepare for future epidemic events. We provide the first exhaustive electronic health record analysis of the over 100,000 COVID-related hospitalisations which took place in the region of Lombardy throughout the year 2020 and assess the monthly evolution, risk factors and outcomes of disease in the hospitalised population of one of the most severely affected regions in Europe.

## Methods

### Data

We analysed de-identified hospitalisation data from the DB-COVID-19 database provided to us by the region of Lombardy. This database included detailed clinical information extracted from the electronic health records of 92,172 COVID-19 patients for a total of 113,145 hospitalisations, covering the period between 1 February 2020 and 30 December 2020. According to the standard protocol adopted in Italian hospitals, including hospitals in Lombardy, patients could either self-refer or be transferred to the emergency room by ambulance, depending on the severity of clinical presentation and COVID-19 related symptoms. Before entering the emergency room, a first evaluation (triage) is carried out by a nurse, who assigns the patient a colour code, based on the severity of the clinical symptoms (white, green, yellow, or red) to establish the priority of access to treatment. Only the white code, which represents a situation of no clinical urgency, has a fixed treatment fee of 25 euros. The other codes and related treatments are fully covered by the national health system and free for the patient in both public and privately owned hospitals accredited by the national health system (hereafter referred to as private hospitals).

The DB-COVID-19 database is composed of a series of datasets containing personal and demographic information (including age, sex, year of birth and province of residence), information on vital signs and clinical status, comorbidities (described below), vaccination status, location (municipality and name of the hospital) and type of hospitalisation (including type of ward and ICUs), number of positive COVID-19 swabs, and serological test results. Data on ethnicity and socio-economic status were not available for extraction in the database.

### Data Analysis

Each of the datasets included in the DB-COVID-19 database features an anonymised personal identification code that uniquely identifies each patient and permits the harmonisation and linkage of data across records. We estimated patient age using the year of birth and the date of patients’ first COVID-19-related hospitalisation; we then grouped patients into 10-year age bands (and also created a dichotomous age variable <70/≥70 years), to be included in our analyses. We then reviewed patients’ pre-existing conditions and classified comorbidities by number (none, 1, 2 or >2 comorbidities) and by type, computing the following categories: diabetes mellitus (including types 1 and 2, complicated and uncomplicated clinical course), hypertension, cardiometabolic diseases (including hypercholesterolaemia, arrhythmic and non-arrhythmic heart disease, venous, arterial and cerebral vascular disease, heart failure, ischaemic heart disease, valvular heart disease), tumours (including active neoplastic disease, remission and follow up to treatment), respiratory diseases (including respiratory failure, asthma, COPD), HIV and other forms of immunosuppression (including HIV, active and inactive transplants) and renal diseases (including patients with chronic renal failure and those undergoing dialysis). We decided to include diabetes mellitus and hypertension as separate covariates in our analyses to reflect their importance as independent predictors of COVID-19-related outcomes, which has been previously reported in the scientific literature (and is described further in the Discussion). We also classified hospitals into public or private (privately owned but accredited by the Italian National Health Service (NHS) and freely accessible to all citizens) providers and as primary or secondary hospitals, depending on size and basing ourselves on classifications provided by regional Public Health authorities.

We stratified the total number of hospitalisations and deaths by month, age, sex, and type of hospital (public vs. private). Finally, case-fatality rate (CFR) was computed as the number of total deaths per total number of hospitalisations.

Overall survival (OS) was defined as time, expressed in days, from the first COVID-19 hospitalisation to the date of death and was estimated using the Kaplan Meier (KM) method. For survivors, the follow up period lasted until the end of the study, i.e., 31 December 2020. We also calculated the median follow-up period for patients. The effect of potential predictors on OS was estimated using multivariate Cox proportional hazards models, calculating hazard ratios (HRs) and corresponding 95% confidence intervals (CIs). The crude HRs were also computed and displayed in Supplementary Table S1 of the Supplementary Material. We tested the proportional hazard condition for all variables, one at time, including in the model interactions between covariates and the logarithmic function of survival time, and we also tested these new variables together. Since the overall test of proportionality, as well as the test for most variables, showed significant *p*-values, we decided to adjust for interactions with time in the model. The multivariate model included terms for age in 10-year age bands, sex, period of hospitalisation (February–April/May–July/August–October/November–December), as well as the aforementioned comorbidities, and type of hospital (private/public and primary/secondary). The covariates were evaluated at the time of first hospitalisation. Overall survival and the multivariate analysis were also stratified by first (February–September) and second pandemic wave period (October–December); the logrank test was used to evaluate differences in OS between strata. The results pertaining to these analyses are displayed in the Supplementary Material, Supplementary Table S2 and Supplementary Figure S1. Finally, we estimated the effect of the number of comorbidities (0/1/2/>2) on mortality through a multivariate cox model (Supplementary Table S3).

## Results

Our study population consisted of all patients hospitalised with a SARS-CoV-2 infection in Lombardy during the period of February to December 2020. After checking for data inconsistencies, missing entries and duplicates (*n* = 2552), we obtained a total sample of 110,593 COVID-19-related hospitalisations, referring to a total of 91,851 COVID-19 patients. The median age (± standard deviation SD) of the study population was 65.9 years (±18.5) and 57.8% were male. The vast majority of patients were hospitalised only once with a mean number of 1.2 hospitalisations; the maximum number of hospitalisations per single patient was 10.

### Monthly Distribution of Hospitalisations and Mortality

**FIGURE 1:**
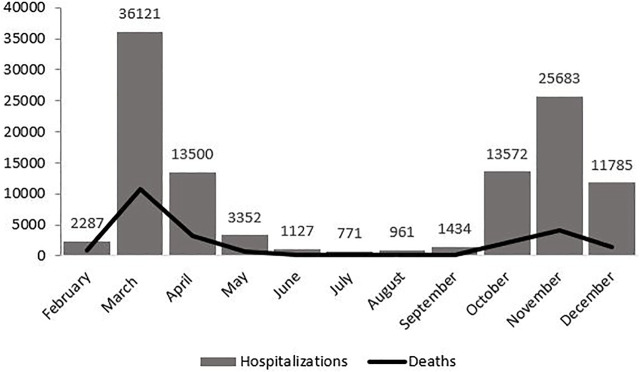
Hospitalisations and deaths trend (Milan, Italy, 2021).

**FIGURE 2:**
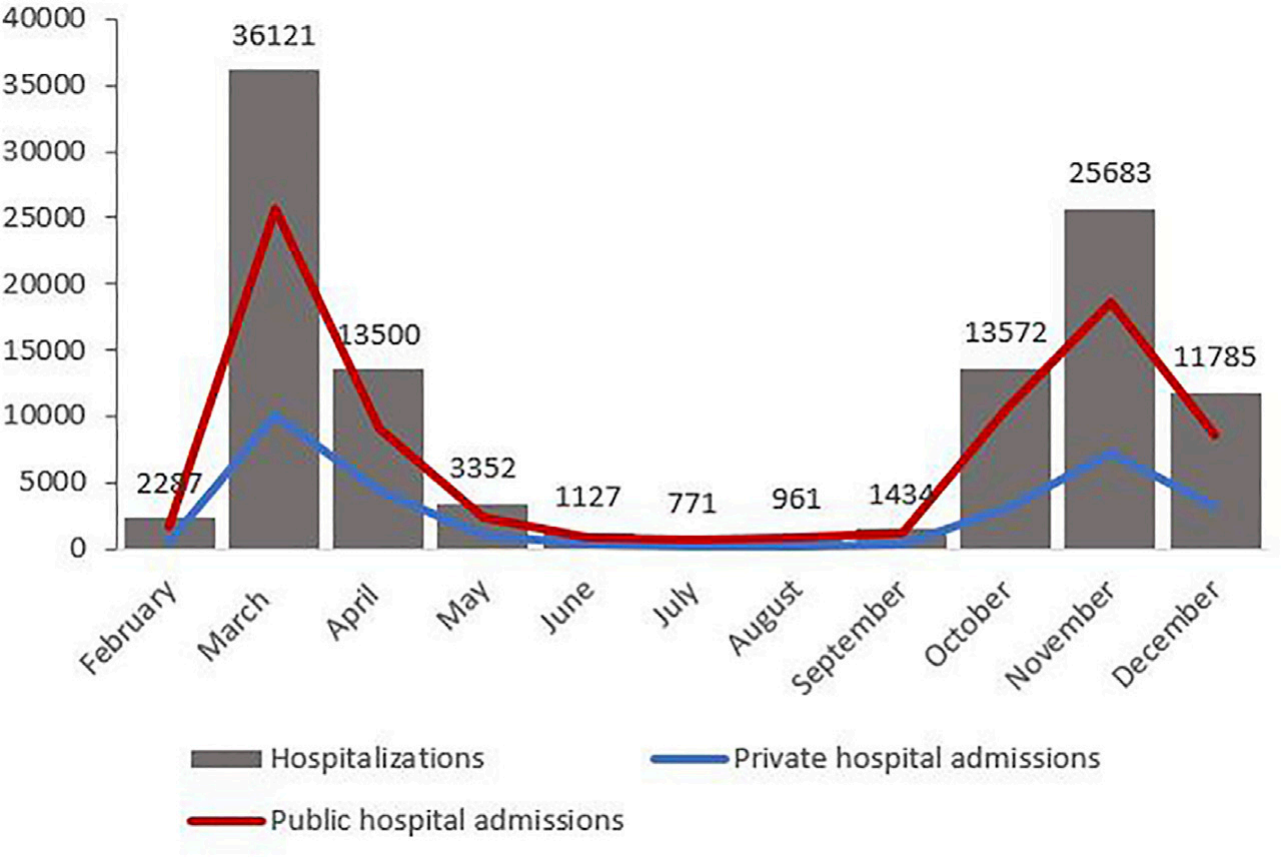
Hospitalisations and type of hospital admission trend (Milan, Italy, 2021).

Table 1 displays the monthly distribution of hospitalisations, deaths and corresponding CFRs, as well as the percentage of people aged 70 years and older among the hospitalised population for each month and the number of admissions per month to private hospitals. Figure 1 displays the mortality curve superimposed on the overall monthly distribution of hospital admissions, while Figure 2 illustrates the monthly distribution of hospitalisations, including the admission trends observed in public and private hospitals separately. Both figures show peaks corresponding to the two epidemic waves observed in March 2020 at 36 121 (33% of total hospitalisations) and in November 2020 at 25 683 hospitalisations (23% of the total hospitalisations). Throughout the study period, the pattern of deaths was consistent with that of hospitalisations, peaking in March and November 2020, and the highest CFR was registered in February (39.8%), followed by March (30%) and April (23.9%) (see Figure 1 and Table 1). Admissions to both public and private hospitals followed this trend, peaking in March (public: 26,020, private: 10,101) and November 2020 (public: 18,526, private: 7,175). Throughout 2020, private hospitals admitted between 20 and 30% of all COVID-related hospitalisations (see Figure 2 and Table 1). Case-fatality in private hospitals essentially overlapped with the overall observed CFR (including public hospitals) (data not shown). Importantly, the prevalence of people aged 70 years and older among the total number of monthly hospitalisations was high throughout the year, peaking at 63% in February and reaching its lowest value at 38.5% in August 2020 (Table 1).

**TABLE 1:** Monthly distribution of hospitalisations (overall and to private hospitals), deaths, case-fatality rate and the proportion of hospitalised patients aged 70 + (Milan, Italy, 2021).

Month	Hospital admissions	Deaths	CFR (%)	Patients aged 70+ (%)	Private hospital admissions
February	2287	910	39.8	63.1	527
March	36121	10849	30	48.7	10101
April	13500	3232	23.9	52.4	4411
May	3352	755	22.5	53.8	1034
June	1127	235	20.9	54.1	294
July	771	122	15.8	48.6	155
August	961	123	12.8	38.5	125
September	1434	185	12.9	39.0	267
October	13572	2204	16.2	42.9	2975
November	25683	4179	16.3	51.9	7157
December	11785	1402	11.9	55.9	3230

### Hospitalisations and Mortality by Comorbidity

**FIGURE 3:**
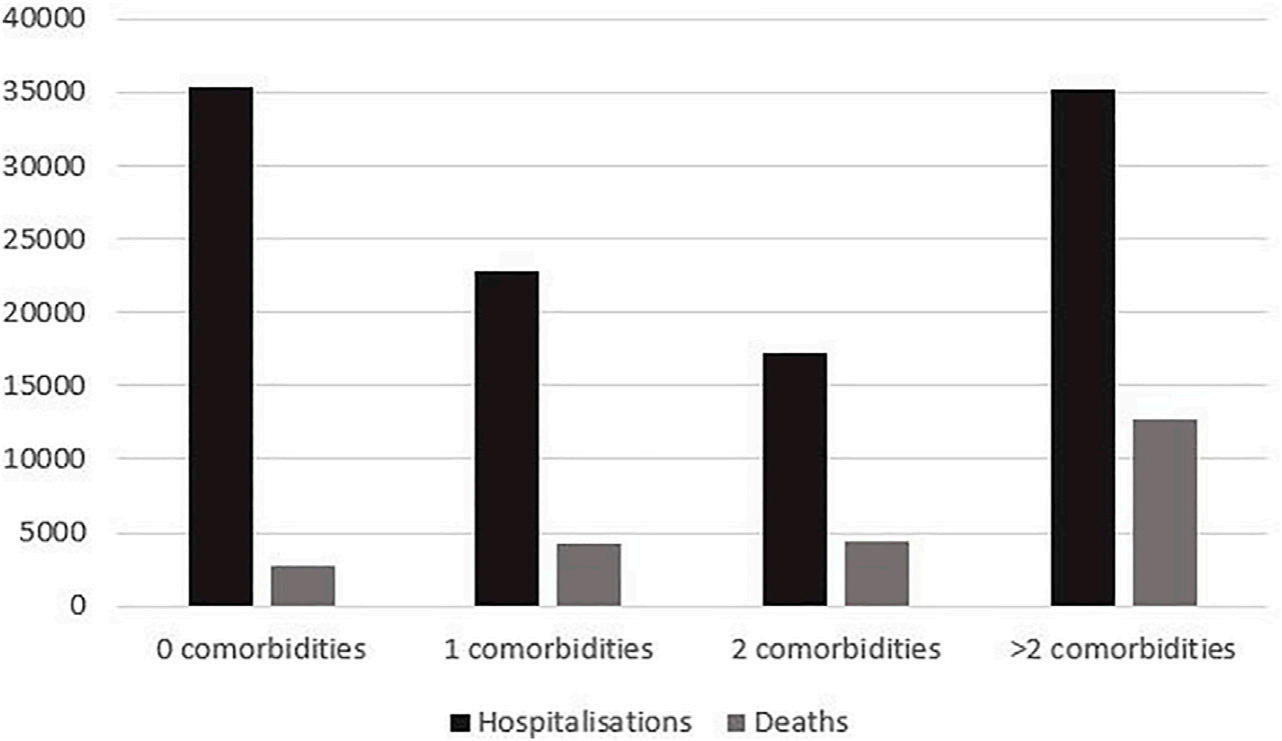
Distribution of hospitalisations and deaths by number of comorbidities (Milan, Italy, 2021).

Figure 3 displays the distribution of hospitalisations and deaths stratified by number of comorbidities. Approximately 1 in 2 hospitalisations occurred in patients without or affected by only one chronic condition. CFR ratio was higher in patients hospitalised with one chronic condition, than in individuals without comorbidities (CFR 18.7% and 7.9%, respectively) and having two or more chronic condition was associated with a CFR of 61.9% (data not shown). The most frequent chronic conditions in all hospitalisations retrieved in the analysed database were hypertension (25.3%), myocardiopathies (11.2%), hypercholesterolaemia (9.8%) and diabetes mellitus type II (8%). Overall, 82% of the hospitalised population were affected by some form of cardiovascular condition, while 30% suffered from a metabolic disease such as diabetes or hypercholesterolaemia.

### Distribution of Hospitalisations and Deaths by Age, Sex, Period, Chronic Condition, and Hospital Characteristics

Table 2 displays the distribution of hospitalisations and deaths according to selected baseline characteristics and type of hospital, including adjusted HRs and corresponding 95% CIs. The median follow-up of patients was 62 days. With reference to patients ≤30 years old, risk of death increased progressively with each successive 10-year age band [age 30–40 HR: 1.24 (0.77–2.01), age 40–50 HR: 3.60 (2.44–5.31), age 50–60 HR: (8.11 5.55–11.83), age 60–70 HR: 24.69 (16.82–36.26), age 70–80 HR: 58.10 (39.14–86.22), age 80–90 HR: 106.68 (71.01–160.27), age ≥90 HR: 180.96 (118.80–275.64)]. Overall survival was better in females compared to males [HR 0.60 (95% CI 0.57–0.64)]. Hospitalisations during the months of May to July [HR 0.39 (0.36–0.42)], August to October [HR 0.26 (0.24–0.28)] and November to December 2020 [HR 0.18 (0.17–0.20)] were associated with a lower risk of death compared to the period from February to April 2020, which marked the first wave of the pandemic. The risk of death increased progressively with the number of comorbidities, from 1.27 (95% CI 1.20–1.35) in patients with one condition to 1.44 (95% CI 1.33–1.55) in those with two and to 1.73 (95% CI 1.58–1.90) in those with three or more conditions, as opposed to those without comorbidities (Supplementary Table S3). Type of comorbidity was a less important predictor of risk of death (see Table 2), with moderately elevated hazard ratios detected for diabetes [HR: 1.24 (1.15–1.33)], hypertension [HR: 1.23 (1.15–1.32)] and cardiometabolic conditions excluding hypertension and diabetes [HR: 1.14 (1.06–1.22)]. Having been first hospitalised in a private hospital, as opposed to being hospitalised in a public one, was associated with a slightly lower risk of death [HR 0.67 (95% CI 0.62–0.72)]. Similarly, there appeared to be a weak protective effect of first admittance to a primary hospital on risk of death [HR 0.84 (95% CI 0.78–0.90)], as opposed to first admittance to a secondary hospital (see Table 2). Supplementary Table S2 shows the corresponding adjusted HRs stratified by pandemic wave periods (I wave and II wave); HRs in the two strata were consistent with the HRs calculated over the entire study period.

**TABLE 2:** Hazard ratios and corresponding 95% confidence intervals for risk of death according to selected baseline characteristics and type of hospital. Multivariate analysis (Milan, Italy, 2021).

	N–number of subjects	Deaths (row %)	Adjusted HRs^a^ (95% CIs)
Age			
≤30	4592	67 (1.46)	*1 (Ref)*
30–40	4529	39 (0.86)	1.24 (0.77–2.01)
40–50	8419	207 (2.46)	3.60 (2.44–5.31)
50–60	14705	804 (5.47)	8.11 (5.55–11.83)
60–70	16022	2540 (15.85)	24.69 (16.82–36.26)
70–80	20820	6616 (31.78)	58.10 (39.14–86.22)
80–90	18822	8648 (45.95)	106.68 (71.01–160.27)
>90	3942	2299 (58.32)	180.96 (118.80–275.64)
Sex			
Male	53101	13248 (25.0)	*1 (Ref)*
Female	38750	7972 (20.6)	0.60 (0.57–0.64)
Period			
February–April	44327	13570 (30.61)	*1 (Ref)*
May–July	3297	698 (21.17)	0.39 (0.36–0.42)
August–October	14031	2203 (15.70)	0.26 (0.24–0.28)
November–December	30196	4749 (15.73)	0.18 (0.17–0.20)
Diabetes Mellitus			
No	77563	16346 (21.1)	*1 (Ref)*
Yes	14288	4874 (34.1)	1.24 (1.15–1.33)
Hypertension			
No	47693	6390 (13.4)	*1 (Ref)*
Yes	44158	14830 (33.6)	1.23 (1.15–1.32)
Cardiometabolic diseases			
No	60678	9510 (15.7)	*1 (Ref)*
Yes	31173	11710 (37.6)	1.14 (1.06–1.22)
Tumours/Oncologic diseases			
No	77491	16056 (20.7)	*1 (Ref)*
Yes	14360	5164 (36.0)	1.00 (0.93–1.07)
Respiratory diseases			
No	83346	18311 (22.0)	*1 (Ref)*
Yes	8505	2909 (34.2)	0.96 (0.88–1.05)
HIV and other forms of immunosuppression			
No	90896	20992 (23.1)	*1 (Ref)*
Yes	955	228 (23.9)	1.24 (0.92–1.68)
Renal diseases			
No	86745	18824 (21.7)	*1 (Ref)*
Yes	5106	2396 (46.9)	1.14 (1.04–1.26)
Type of hospital			
Public	69948	15864 (22.7)	*1 (Ref)*
Private	21903	5356 (24.5)	0.67 (0.62–0.72)
Size of hospital			
Secondary	64557	15501 (24.0)	*1 (Ref)*
Primary	27294	5719 (21.0)	0.84 (0.78–0.90)

a The model was mutually adjusted and corrected also for interaction between covariates and logarithmic function of survival time.

### Kaplan-Meier Curve of Overall Survival

**FIGURE 4:**
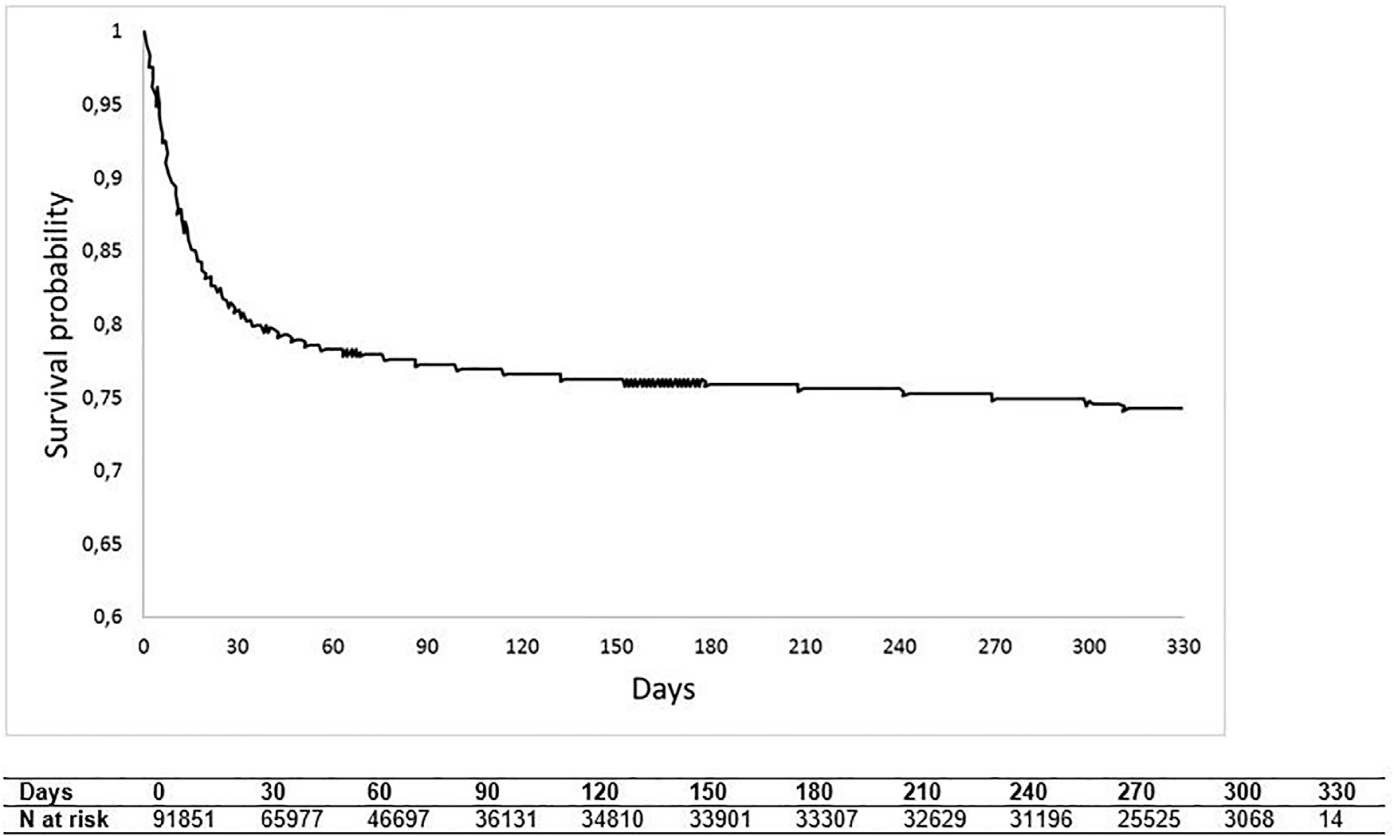
Kaplan-Meier curve of overall survival (Milan, Italy, 2021).

Overall survival (displayed in Figure 4) fell steeply during the first month of hospitalisation, reaching around 80% of survival probability, to then, as time progresses, decline more slowly, reaching at the end of follow-up a probability value of approximately 75%. Supplementary Figure S1 shows OS estimates stratified by pandemic wave periods (I wave and II wave); 1 month after hospitalisation, OS was approximately 85% for the second pandemic wave and 75% for the first pandemic wave (log-rank *p*-value < 0.0001).

## Discussion

Our study found that between February and December 2020, over 91,000 people were hospitalised for COVID-19 across the region of Lombardy. Over 50,000 hospitalisations were registered during the most critical months of both the first and second pandemic waves alone. However, CFRs were considerably higher at the height of the first wave. Strikingly, close to 1 in 2 deaths among hospitalised COVID-19 patients in Lombardy occurred in the month of March 2020 alone. November was the month with the second highest rate of hospitalisations and mortality. Risk of death increased progressively with age and the number of comorbidities, while correlation with type of comorbidity was less pronounced.

Our results are in line with previous evidence: Scortichini et al. estimate that between 15 February and 15 May 2020, Lombardy experienced 25,782 excess deaths, which along with two other northern regions (Veneto and Emilia-Romagna), account for 71.0% of the total excess mortality observed in Italy [47,490, 95% CI (43 984–50 362)] [22]. During the first epidemic wave, Italy displayed the third highest excess mortality rate in Europe (740 excess deaths per million inhabitants) closely following Spain and the United Kingdom (950 excess deaths per million inhabitants) [21]. Consistent with other European settings, the peak of hospitalisations in Lombardy was observed in March, which registered one-third of all COVID-19 hospitalisations in 2020 and reflects the substantial struggle to contain the spread of disease during the first epidemic wave. Many European countries faced the initial phases of the pandemic with outdated and inadequate pandemic preparedness plans, which had been primarily designed to manage influenza epidemics in response to recommendations issued by the World Health Organization in 2006 [23]. Scarce testing capacities and the stagnant procurement of masks and other personal protective equipment further hindered an effective response and forced countries to apply stringent containment measures with substantial long-term economic, social and Public Health consequences [21]. Beyond this, the Italian context presents two major inherent idiosyncrasies: firstly, a combination of population sociodemographic characteristics, which determined a higher disease susceptibility and which we already presented in the introduction, and secondly, having been the first European region to be hit by the virus, which allowed less time to develop and implement comprehensive policy responses [24]. It is estimated that circulation of the virus in the Italian population started several weeks before the discovery of the first native case on 21st February. This reduced the immediate effectiveness of containment measures and lockdown, which represented the primary government response in light of the limited resources to face the sudden health crisis [23, 25]. As a result, hospitals across Lombardy, considered as having one of the best-equipped regional health systems in Italy, reported oversaturation with a severe shortage of hospital beds, ventilators and health professionals within weeks [26]. It appears that hospital saturation was directly proportional to risk of mortality, underlining the importance of equipping health systems to mount rapid responses and redirect resources in moments of need, including recruiting and training health care workers, sourcing hospital beds to expand hospital capacities and contracting medical countermeasures through flexible supply chains.

Throughout both epidemic waves, Lombardy established a hospital network for managing and absorbing the constant demand for hospital beds from COVID-19 admissions through the creation of a hospital hub-and-spoke model. Hub hospitals exclusively encompassed primary hospitals, since they are equipped with infectious disease departments and large emergency departments (classified as either CTS—highly specialised trauma centres or CTZ—Referral trauma centres within specific geographical areas). It is possible that the expertise and advanced equipment of primary structures may have had a protective effect on patient outcomes. Conversely, public structures have been historically underfunded (as discussed in the Introduction) and thus may have been slightly less geared to manage COVID-19 patients than private structures, contributing to the slightly elevated risk of mortality we observed in public hospitals. However, since we only detected small differences across private/public and primary/secondary centres, this should be interpreted with caution and warrants further analyses to untangle the specific interactions between size and type of hospital in determining COVID-19 outcomes.

Over 50% of all COVID-related hospitalisations occurred in patients aged 70 years and older. Furthermore, our results suggest an up to 180 times higher risk of death in this patient group compared to younger hospitalised patients. This confirms previous findings in the literature reporting an exponential increase in cases and deaths with age [27, 28] and underscores the increased vulnerability of older populations to developing a more serious form of the disease requiring specialist medical attention [29]. Various theories have attempted to explain the steep age gradient observed in COVID-19 susceptibility and mortality, from the most logical explanation that immune system response decreases with age to possible cross-reactivities with other viruses inferring immunological memory in younger patients with more social contacts [27, 30]. In this context, nursing homes appear to have played a crucial role in aggravating mortality trends in Northern Italy. Notably, Italian care home facilities do not have the status of medical facilities and have been severely understaffed since the last economic crisis. In the early stages of the pandemic, when resources were lacking and few policies had been implemented, these facilities did not benefit from a timely provision of personal protective equipment, emergency care equipment or stringent containment measures to reduce the contact with visitors and staff [28, 31]. In Lombardy, the decision by regional authorities to allocate sub-acute COVID-19 patients to some nursing homes in an effort to alleviate the pressure on hospitals substantially may have worsened the situation and resulted in immediate outbreaks among vulnerable populations within these facilities [32]. A modelling study by Ciminelli et al. on Lombardy estimates that in those municipalities, in which more than 10% of the elderly population resided in nursing homes, COVID-19 mortality was twice as high as opposed to localities without intown nursing homes [28]. At European level, over 90% of COVID-19 deaths recorded during the first and second epidemic waves occurred in people over the age of 60 years and in those affected by chronic conditions [21]. Aside from the demonstrated risks related to higher age, there appears to be a direct, and possibly synergistic, correlation between having pre-existing comorbidities (such as diabetes, chronic obstructive pulmonary disease, cardiovascular diseases, hypertension, malignancies, HIV and others) and the probability of developing a severe form of COVID-19. In our sample, the CFR in patients without comorbidities was 7.9%, as opposed to 18.7% in patients with only one comorbidity and a staggering 62% in patients with two or more comorbidities. Further confirmed by the findings in our survival analysis, the number of comorbidities appears to be directly proportional to an increase in the risk of dying from a severe form of disease. As Gold et al. previously reported, severe COVID cases report a higher prevalence of hypertension and type 2 diabetes [33]: in our database, the prevalence of these two conditions was 25.3% and 9.8%, respectively. Importantly, 82% of our studied hospitalised population suffered from some form of chronic pre-existing condition. Interestingly, the risk of death related to number of comorbidities was more pronounced than the risk related to individual comorbidities such as diabetes mellitus, hypertension and other cardiometabolic diseases, in our patient sample. Although these diseases were separately associated with a higher risk of mortality, there may be an additive effect with an increasing number of comorbidities.

It emerges that lack of preparedness had dramatic effects on Public Health, generating a burden of excess deaths, which could have been prevented through previous experience in managing epidemics, a more rapid and better coordinated pandemic response and targeted policies tailored at catering to the specific needs of vulnerable populations. Questionable policies implemented in nursing homes or lack thereof cost thousands of elderly people their lives. More generally, COVID-19 has brought to light profound pre-existing care gaps in European health systems, including the Italian NHS. In the years prior to the pandemic, the number of hospital beds in Italy had progressively decreased by approximately 30% between 2000 and 2017 as a result of cost-containment measures, plummeting to 3 beds per 1000 inhabitants, compared to the European average of 5 hospital beds [23]. Similarly, the number of ICU beds was well-below the European average (8.6 ICU beds vs. 12.9 ICU beds/100,000 population) [21]. Beyond this, the pandemic has highlighted the need for resilient preventive and primary health care systems to prevent the saturation of hospitals in times of emergency and forestall the long-term effects of lifestyle risk factors and non-communicable diseases (NCDs) on population health. A serious issue, which will accompany the post-pandemic economic and societal recovery, is represented by the interruption of these services, elective medical procedures and NCD screening programmes throughout the duration of the pandemic [34]. In view of future cross-border health threats, health systems should become more resilient to external shocks and develop solutions to guarantee essential services in times of emergency. This may partly be achieved through innovative solutions such as telemedicine services, eHealth and remote health care. It will be of equal importance for Italy, and in particular Lombardy, to take advantage of the vast amount of data collected and experience gained to establish guidelines on the effective management of pandemics, not only in the hospital and health care facility setting, but also more broadly at regional and national level. Administrative databases, such as the one we used for this study, have allowed researchers also in other national contexts to uncover epidemiological patterns and inform both academic and policy worlds to better shape their COVID-19 responses in view of a possible soaring of cases and further pandemic waves [35, 36]. These elements tie into the lessons learned from COVID and into an overarching strategy, which is starting to emerge in political agendas across Europe, of better seizing digital technologies and Big Data sources for Public Health objectives to strengthen the resilience of health systems in view of future emergencies.

Our study has a number of limitations mainly related to the intrinsic constraints of studies using administrative data. Specifically, our dataset counted approximately 2,500 duplicates or entries with missing data, which had to be excluded from analysis. Secondly, we do not know whether patients included in our sample were hospitalised for COVID-related reasons or whether they were first tested positive after their hospitalisation. Hence, we do not know the proportion of patients who were originally hospitalised for conditions other than COVID-19. A third limitation lies in the breadth of indicators collected within the DB-database, which lacks information about patients’ socioeconomic background, ethnicity, as well as more comprehensive health parameters, including information on ICU admittance, limiting our ability to characterise and draw further conclusions on the biopsychosocial risk factors associated to COVID-related hospitalisations in Lombardy.

However, despite these limitations, the current study provide a comprehensive overview of over 100,000 COVID-related hospitalisations in Lombardy, being one of the largest database used to assess COVID-19 trends and mortality during the first two epidemic waves in Italy. Our findings highlighted a significantly higher risk of dying in older hospitalised patients and in those patients affected by one or more chronic comorbidities, underlining the urgent need to better protect vulnerable population groups, which make up increasingly large proportions of European populations as a result of the ongoing demographic and epidemiological transitions. While strengthening the evidence currently available about features of patients at risk of severe COVID-19, we also provide novel insights into the Italian pandemic scenario, uncovering the mechanisms of the crisis within the regional health system of Lombardy and, on a larger scale, the European response as a whole. More broadly, these findings can inform future policy and response mechanisms in view of newly emerging COVID-19 variants and the potential emergence of novel pathogens.
