# Supplementary material for: The First 110,593 COVID-19 Patients Hospitalised in Lombardy: A Regionwide Analysis of Case Characteristics, Risk Factors and Clinical Outcomes
Source: Int J Public Health. 2022 May 11;67:1604427. doi: 10.3389/ijph.2022.1604427 (PMC9131487; doi:10.3389/ijph.2022.1604427)
Supplement: Supplementary file 1 [file DataSheet1.docx]

**SUPPLEMENTARY MATERIAL**

**Table S1.** Hazard ratios and corresponding 95% confidence intervals for risk of death according to selected baseline characteristics and type of hospital. Univariate analysis. (Milan, Italy. 2021)

|  | **N – number of subjects** | **Deaths (row %)** | **Crude HRs^a^ (95% CIs)** |
| --- | --- | --- | --- |
| **Age** |  |  |  |
| ≤30 | 4592 | 67 (1.46) | *1 (Ref)* |
| 30-40 | 4529 | 39 (0.86) | 1.26 (0.78-2.04) |
| 40-50 | 8419 | 207 (2.46) | 3.87 (2.64-5.68) |
| 50-60 | 14705 | 804 (5.47) | 9.12 (6.30-13.22) |
| 60-70 | 16022 | 2540 (15.85) | 29.66 (20.40-43.12) |
| 70-80 | 20820 | 6616 (31.78) | 70.73 (48.25-103.69) |
| 80-90 | 18822 | 8648 (45.95) | 120.96 (81.69-179.11) |
| >90 | 3942 | 2299 (58.32) | 173.89 (116.05-260.57) |
|  |  |  |  |
| **Sex** |  |  |  |
| Male | 53101 | 13248 (25.0) | *1 (Ref)* |
| Female | 38750 | 7972 (20.6) | 0.68 (0.64-0.72) |
| **Period** |  |  |  |
| February-April | 44327 | 13570 (30.61) | *1 (Ref)* |
| May-July | 3297 | 698 (21.17) | 0.42 (0.39-0.45) |
| August-October | 14031 | 2203 (15.70) | 0.23 (0.21-0.25) |
| November-December | 30196 | 4749 (15.73) | 0.20 (0.18-0.21) |
| **Diabetes Mellitus** |  |  |  |
| No | 77563 | 16346 (21.1) | *1 (Ref)* |
| Yes | 14288 | 4874 (34.1) | 1.89 (1.75-2.02) |
| **Hypertension** |  |  |  |
| No | 47693 | 6390 (13.4) | *1 (Ref)* |
| Yes | 44158 | 14830 (33.6) | 3.07 (2.88-3.28) |
| **Cardiometabolic diseases** |  |  |  |
| No | 60678 | 9510 (15.7) | *1 (Ref)* |
| Yes | 31173 | 11710 (37.6) | 2.64 (2.49-2.81) |
| **Tumours/Oncologic diseases** |  |  |  |
| No | 77491 | 16056 (20.7) | *1 (Ref)* |
| Yes | 14360 | 5164 (36.0) | 1.61 (1.51-1.73) |
| **Respiratory diseases** |  |  |  |
| No | 83346 | 18311 (22.0) | *1 (Ref)* |
| Yes | 8505 | 2909 (34.2) | 1.46 (1.34-1.59) |
|  |  |  |  |
| **HIV and other forms of immunosuppression** |  |  |  |
| No | 90896 | 20992 (23.1) | *1 (Ref)* |
| Yes | 955 | 228 (23.9) | 0.78 (0.58-1.06) |
| **Renal diseases** |  |  |  |
| No | 86745 | 18824 (21.7) | *1 (Ref)* |
| Yes | 5106 | 2396 (46.9) | 2.09 (1.90-2.29) |
| **Type of hospital** |  |  |  |
| Public | 69948 | 15864 (22.7) | *1 (Ref)* |
| Private | 21903 | 5356 (24.5) | 0.86 (0.80-0.92) |
| **Size of hospital** |  |  |  |
| Secondary | 64557 | 15501 (24.0) | *1 (Ref)* |
| Primary | 27294 | 5719 (21.0) | 0.77 (0.72-0.82) |

^a^ The models were corrected also for interaction between covariates and logarithmic function of survival time.

**Table S2.** Hazard ratios and corresponding 95% confidence intervals for risk of death according to selected baseline characteristics and type of hospital and in strata of I pandemic wave and II pandemic wave. Multivariate analysis. (Milan, Italy. 2021)

|  | **N – number of subjects** | **Deaths (row %)** | **I wave**  **Adjusted HRs^a^ (95% CIs)** | **II wave**  **Adjusted HRs^a^ (95% CIs)** |
| --- | --- | --- | --- | --- |
| **Age** |  |  |  |  |
| ≤30 | 4592 | 67 (1.46) | *1 (Ref)* | *1 (Ref)* |
| 30-40 | 4529 | 39 (0.86) | 1.66 (0.87-3.18) | 1.55 (0.65-3.69) |
| 40-50 | 8419 | 207 (2.46) | 4.82 (2.77-8.38) | 4.74 (2.36-9.50) |
| 50-60 | 14705 | 804 (5.47) | 9.90 (5.74-17.05) | 14.95 (7.63-29.30) |
| 60-70 | 16022 | 2540 (15.85) | 28.77 (16.62-49.79) | 53.84 (26.81-108.09) |
| 70-80 | 20820 | 6616 (31.78) | 64.23 (36.78-112.16) | 150.90 (72.55-313.85) |
| 80-90 | 18822 | 8648 (45.95) | 106.10 (60.13-187.20) | 350.99 (162.44-758.38) |
| >90 | 3942 | 2299 (58.32) | 162.81 (91.13-290.88) | 709.09 (315.62-1593.08) |
|  |  |  |  |  |
| **Sex** |  |  |  |  |
| Male | 53101 | 13248 (25.0) | *1 (Ref)* | *1 (Ref)* |
| Female | 38750 | 7972 (20.6) | 0.55 (0.51-0.60) | 0.74 (0.65-0.84) |
| **Diabetes Mellitus** |  |  |  |  |
| No | 77563 | 16346 (21.1) | *1 (Ref)* | *1 (Ref)* |
| Yes | 14288 | 4874 (34.1) | 1.23 (1.14-1.34) | 1.32 (1.13-1.53) |
| **Hypertension** |  |  |  |  |
| No | 47693 | 6390 (13.4) | *1 (Ref)* | *1 (Ref)* |
| Yes | 44158 | 14830 (33.6) | 1.29 (1.19-1.40) | 1.12 (0.96-1.30) |
| **Cardiometabolic diseases** |  |  |  |  |
| No | 60678 | 9510 (15.7) | *1 (Ref)* | *1 (Ref)* |
| Yes | 31173 | 11710 (37.6) | 1.12 (1.04-1.21) | 1.18 (1.03-1.36) |
| **Tumours/Oncologic diseases** |  |  |  |  |
| No | 77491 | 16056 (20.7) | *1 (Ref)* | *1 (Ref)* |
| Yes | 14360 | 5164 (36.0) | 0.97 (0.90-1.05) | 1.10 (0.95-1.28) |
| **Respiratory diseases** |  |  |  |  |
| No | 83346 | 18311 (22.0) | *1 (Ref)* | *1 (Ref)* |
| Yes | 8505 | 2909 (34.2) | 0.91 (0.82-1.00) | 1.21 (1.01-1.44) |
| **HIV and other forms of immunosuppression** |  |  |  |  |
| No | 90896 | 20992 (23.1) | *1 (Ref)* | *1 (Ref)* |
| Yes | 955 | 228 (23.9) | 1.20 (0.86-1.68) | 1.49 (0.74-3.01) |
| **Renal diseases** |  |  |  |  |
| No | 86745 | 18824 (21.7) | *1 (Ref)* | *1 (Ref)* |
| Yes | 5106 | 2396 (46.9) | 1.08 (0.96-1.21) | 1.44 (1.18-1.76) |
| **Type of hospital** |  |  |  |  |
| Public | 69948 | 15864 (22.7) | *1 (Ref)* | *1 (Ref)* |
| Private | 21903 | 5356 (24.5) | 0.65 (0.60-0.70) | 0.84 (0.71-0.99) |
| **Size of hospital** |  |  |  |  |
| Secondary | 64557 | 15501 (24.0) | *1 (Ref)* | *1 (Ref)* |
| Primary | 27294 | 5719 (21.0) | 0.71 (0.65-0.77) | 0.94 (0.89-1.00) |

^a^ The models were adjusted and corrected also for interaction between covariates and logarithmic function of survival time.

**Table S3.** Hazard ratios and corresponding 95% confidence intervals for risk of death according to selected baseline characteristics (including number of comorbidities) and type of hospital. Multivariate analysis. (Milan, Italy. 2021)

|  | **N – number of subjects** | **Deaths (row %)** | **Adjusted HRs (95% CI)** |
| --- | --- | --- | --- |
| **Age** |  |  |  |
| ≤30 | 4592 | 67 (1.46) | *1 (Ref)* |
| 30-40 | 4529 | 39 (0.86) | 1.23 (0.76-1.99) |
| 40-50 | 8419 | 207 (2.46) | 3.44 (2.33-5.08) |
| 50-60 | 14705 | 804 (5.47) | 7.46 (5.11-10.88) |
| 60-70 | 16022 | 2540 (15.85) | 21.90 (14.92-32.15) |
| 70-80 | 20820 | 6616 (31.78) | 50.62 (34.12-75.10) |
| 80-90 | 18822 | 8648 (45.95) | 92.68 (61.73-139.15) |
| >90 | 3942 | 2299 (58.32) | 157.92 (103.78-240.31) |
|  |  |  |  |
| **Sex** |  |  |  |
| Male | 53101 | 13248 (25.0) | *1 (Ref)* |
| Female | 38750 | 7972 (20.6) | 0.60 (0.57-0.64) |
|  |  |  |  |
| **Period** |  |  |  |
| February-April | 44327 | 13570 (30.61) | *1 (Ref)* |
| May-July | 3297 | 698 (21.17) | 0.39 (0.36-0.42) |
| August-October | 14031 | 2203 (15.70) | 0.26 (0.24-0.28) |
| November-December | 30196 | 4749 (15.73) | 0.18 (0.17-0.20) |
|  |  |  |  |
| **Comorbidities** |  |  |  |
| 0 | 30109 | 2399 (8.0) | *1 (Ref)* |
| 1 | 18931 | 3709 (19.6) | 1.27 (1.20-1.35) |
| 2 | 14113 | 3932 (27.9) | 1.44 (1.33-1.55) |
| >2 | 28698 | 11180 (39.0) | 1.73 (1.58-1.90) |
| **Type of hospital** |  |  |  |
| Public | 69948 | 15864 (22.7) | *1 (Ref)* |
| Private | 21903 | 5356 (24.5) | 0.67 (0.62-0.72) |
| **Size of hospital** |  |  |  |
| Secondary | 64557 | 15501 (24.0) | *1 (Ref)* |
| Primary | 27294 | 5719 (21.0) | 0.84 (0.78-0.90) |

**Figure S1.** Kaplan-Meier curve of overall survival in strata of I pandemic wave and II pandemic wave. (Milan, Italy. 2021)

| **N at risk/Days** | **0** | **30** | **60** | **90** | **120** | **150** | **180** | **210** | **240** | **270** | **300** | **330** |
| --- | --- | --- | --- | --- | --- | --- | --- | --- | --- | --- | --- | --- |
| **I wave period** | 49627 | 37645 | 36516 | 36093 | 34810 | 33901 | 33307 | 32629 | 31196 | 25525 | 3068 | 14 |
| **II wave period** | 42224 | 28332 | 10181 | 38 |  |  |  |  |  |  |  |  |
